# Frequent upregulation of G9a promotes RelB-dependent proliferation and survival in multiple myeloma

**DOI:** 10.1186/s40164-020-00164-4

**Published:** 2020-05-22

**Authors:** Xi Yun Zhang, Deepa Rajagopalan, Tae-Hoon Chung, Lissa Hooi, Tan Boon Toh, Johann Shane Tian, Masturah Bte Mohd Abdul Rashid, Noor Rashidha Bte Meera Sahib, Mengjie Gu, Jhin Jieh Lim, Wilson Wang, Wee Joo Chng, Sudhakar Jha, Edward Kai-Hua Chow

**Affiliations:** 1 Cancer Science Institute of Singapore, Centre for Translational Medicine, National University of Singapore, (MD6) #13-01G, 14 Medical Drive, Singapore, 117599 Singapore; 2 Department of Medicine, Yong Loo Lin School of Medicine, National University of Singapore, Singapore, 119228 Singapore; 3 The N.1 Institute for Health (N.1), National University of Singapore, Center for Life Sciences, 28 Medical Drive, Singapore, 117456 Singapore; 4 KYAN Therapeutics, Inc., Singapore, Singapore; 5 Department of Pharmacology, Yong Loo Lin School of Medicine, National University of Singapore, Singapore, 117597 Singapore; 6 Department of Orthopaedic Surgery, National University of Singapore, Kent Ridge, Singapore, 119074 Singapore; 7 National University Cancer Institute, National University Health System, Singapore, 119228 Singapore; 8 Department of Biochemistry, National University of Singapore, Singapore, Singapore

**Keywords:** Multiple myeloma, G9a, RelB, NF-κB pathway, Proliferation, Tumorigenesis, Therapeutic target

## Abstract

**Background:**

Multiple myeloma is an incurable hematological malignancy characterized by a heterogeneous genetic and epigenetic landscape. Although a number of genetic aberrations associated with myeloma pathogenesis, progression and prognosis have been well characterized, the role of many epigenetic aberrations in multiple myeloma remain elusive. G9a, a histone methyltransferase, has been found to promote disease progression, proliferation and metastasis via diverse mechanisms in several cancers. A role for G9a in multiple myeloma, however, has not been previously explored.

**Methods:**

Expression levels of G9a/EHMT2 of multiple myeloma cell lines and control cells Peripheral Blood Mononuclear Cells (PBMCs) were analyzed. Correlation of G9a expression and overall survival of multiple myeloma patients were analyzed using patient sample database. To further study the function of G9a in multiple myeloma, G9a depleted multiple myeloma cells were built by lentiviral transduction, of which proliferation, colony formation assays as well as tumorigenesis were measured. RNA-seq of G9a depleted multiple myeloma with controls were performed to explore the downstream mechanism of G9a regulation in multiple myeloma.

**Results:**

G9a is upregulated in a range of multiple myeloma cell lines. G9a expression portends poorer survival outcomes in a cohort of multiple myeloma patients. Depletion of G9a inhibited proliferation and tumorigenesis in multiple myeloma. RelB was significantly downregulated by G9a depletion or small molecule inhibition of G9a/GLP inhibitor UNC0642, inducing transcription of proapoptotic genes *Bim* and *BMF*. Rescuing RelB eliminated the inhibition in proliferation and tumorigenesis by G9a depletion.

**Conclusions:**

In this study, we demonstrated that G9a is upregulated in most multiple myeloma cell lines. Furthermore, G9a loss-of-function analysis provided evidence that G9a contributes to multiple myeloma cell survival and proliferation. This study found that G9a interacts with NF-κB pathway as a key regulator of RelB in multiple myeloma and regulates RelB-dependent multiple myeloma survival. G9a therefore is a promising therapeutic target for multiple myeloma.

## Background

Multiple myeloma (MM) is a neoplastic plasma cell disorder characterized by the abnormal expansion of monoclonal plasma cells in the bone marrow and most frequently diagnosed among people aged 65-74, which accounts for approximately 13% of hematologic malignancies [1]. Increased interest in addressing MM is largely due to growing incidence rates for MM along with an increasingly aging society, as well as the fact that MM remains incurable [2, 3]. Several advances in the treatment of multiple myeloma have improved the 5-year survival rate of MM since the introduction of proteasome inhibitor-bortezomib and immunomodulatory drugs. However, the rapid development of resistance to bortezomib and other MM drugs continues to prevent curative therapeutic options for MM [4, 5]. Prognosis for relapsed and refractory patients is poor, with about only 9 months median overall survival (OS) and 5 months event-free survival [6]. As such, additional molecular targets are needed to improve the armamentarium available for treating MM patients.

A major hurdle in the treatment of MM is the diversity of genetic alterations that drive MM progression, with translocations targeting the *MYC* gene, activating mutations in oncogenes such as *KRAS, NRAS* of the MAPK pathway, loss-of-function mutations in tumor suppressor genes like *P53*, *IRF4* and *PRDM1*, mutations resulting in hyperactivation of NF-κB pathway all reported to having been observed in MM [7–12.] Many of these identified genetic aberrations have been proven difficult to therapeutically target, due to their mechanism of action as well as role in normal biology. Recent successes in the use of epigenetic drugs in MM and other cancers suggest that targeting epigenetic drivers of MM may provide improved treatment options. Epigenetic changes such as aberrant DNA methylation [13–15] and histone modifications [16, 17] have been found to correlate with pathogenesis and progression of MM. Numerous mutations of epigenetic modifiers that contribute to progression and pathogenesis of MM have also been reported [18]. For example, mutations in DNA methyltransferases DNMT3A may be involved in drug resistance and mutations of Class I histone deacetylases (HDAC1) have been associated with poor prognosis in MM [19–21]. Recently, the histone deacetylase inhibitor panobinostat has been approved by FDA for clinical use in MM patients with a number of other epigenetic drugs entering clinical trials for MM [22–24].

Large scale analysis of MM patient samples has revealed a series of mutations and copy number variations (CNVs) of epigenetic modifiers [18, 19, 25]. Among the reported genomic alterations of epigenetic modifiers, copy number amplifications of EHMT2 (euchromatic histone-lysine N-methyltransferase 2), which encodes the histone methyltransferase G9a, were frequently detected in MM [19]. G9a is a SET domain-containing histone methyltransferase that mediates histone H3 lysine 9 (H3K9) mono- and dimethylation, which are essential for transcriptional repression in the euchromatin region during cell differentiation and embryogenesis [26–28]. G9a has shown to play a very limited role in normal B cell development and no role in T cell development, with G9a-deficient mice exhibiting normal lymphocyte development with modest defects in normal B cell proliferation and plasma cell differentiation [29]. Another histone methyltransferase, G9a/G9a-like-protein (GLP), may play a larger role in normal hematological function as GLP-mediated H3K9me2 patterning is associated with lineage commitment in human hematopoietic stem and progenitor cells (HSPCs) and that inhibition with a G9a/GLP inhibitor leads to delayed differentiation [27]. Some of the functions of G9a in cancer may be due to non-histone targets of G9a such as p53, chromatin-remodeling factor Pontin and G9a itself. G9a effects on these non-histone targets were found to be involved in important transcriptional regulations with either a repressive or an activating effect on gene expression by interacting with other histone modifiers or transcriptional factors [30–32]. Moreover, G9a has been found to play a role in carcinogenesis in several cancers including lung cancer [33], head and neck cancer [34], ovarian cancer [31], hepatocellular carcinoma [35] correlating with a poor prognosis. The importance of G9a in various cancer suggests that it might represent a valuable therapeutic target, and several G9a-related inhibitors have demonstrated the potential for therapeutic utility in preclinical cancer studies [36–38]. Several G9a-related inhibitors have been developed and widely used in preclinical studies for novel cancer therapy, however most are not suitable for in vivo or clinical studies due to their poor solubility or broad off-target effects [39]. The G9a/GLP inhibitor UNC0642, however, was designed with an improved pharmacokinetics and high affinity to G9a representing the most suitable tool compound for targeting and inhibiting G9a in preclinical studies [40]. Similar to other G9a-related inhibitors, UNC0642 does still have off-target effects as it can effectively inhibit GLP (IC50 < 2.5 nM) as well as polycomb repressive complex 2-enhancer of zeste 2 (PRC2-EZH2) (IC50 ~ 5 μM). Thus, while there is evidence for the role of G9a in a number of cancers, there are currently no suitable G9a-specific inhibitors for clinical use.

Despite evidence of pro-oncogenic roles for G9a and gains in EHMT2 CNVs, the role of G9a in MM has not been studied. In this study, we investigated the potential role and the underlying mechanisms of G9a in MM. We found that G9a was upregulated in most multiple myeloma cell lines (MMCLs) and patient samples and that knockdown of G9a inhibited proliferation, colony formation of MMCLs in vitro as well as the tumorigenesis of MMCLs in vivo. In line with G9a loss-of-function studies, pharmacological G9a/GLP inhibition with UNC0642 efficiently induced cell death, and significantly reduced proliferation and colony formation of MMCLs. Importantly, we found that G9a is a novel regulator of NF-κB pathway in MM. G9a depletion significantly reduced expression of RelB and p-RelB, and induced transcription of pro-apoptotic factors Bim and BMF, thereby repressing MM cell survival. Since activation of NF-κB pathways, especially the non-canonical NF-κB pathway, has been frequently observed in MM [41], and the RelB-p52 addiction has been found in most subgroups of MM cells [42], the RelB-mediated NF-κB pathway activation by G9a upregulation may serve as an important epigenetic mechanism of MM proliferation and survival, which makes the G9a an important molecular target for NF-κB pathway inhibitors and innovative therapeutic approaches for MM patients.

## Methods

### Cell culture

Human MM cell lines KMS11, KMS12BM, RPMI 8226, Bortezomib-resistant RPMI 8226 (P100v) and JJN3 were kindly provided by Prof. Chng Wee Joo’s lab (Department of Medicine, Cancer Science Institute of Singapore, Yong Loo Lin School of Medicine, National University of Singapore), and have been tested, authenticated and previously used in the peer-reviewed articles produced by the laboratory [43–45]. MM cell lines were maintained in RPMI 1640 medium (BioWest, Kansas City, MO) with supplement of 10% fetal bovine serum (FBS, HyClone) and 1% penicillin/streptomycin (Invitrogen). HEK293T cells (from ATCC, Manassas, VA) were cultured in DMEM high glucose medium (BioWest, Kansas City, MO) supplemented with 10% FBS (HyClone), 1% Pen Strep (Invitrogen). For adherent cell lines, trypsin (Thermo Scientific, cat# 25200-056) was used to dissociate cells each passage. Cryopreserved Peripheral Blood Mononuclear Cells (PBMCs, AllCells) were cultured in RPMI 1640 medium (BioWest, Kansas City, MO) with supplement of 10% FBS (HyClone) and 1% penicillin/streptomycin (Invitrogen) 1 day before pellet collection for whole-cell lysates extraction. All cells were maintained in a humidified atmosphere at 37 °C with 5% carbon dioxide.

### Lentiviral transduction and gene silencing

pLKO.1 vector expressing non-targeting shRNA was purchased from Biocompare with product name TRC Lentiviral shRNA Controls (Dharmacon, # RHS6848). Targeting sequence is: 5′-CCGGTTGGTTTACATGTTGTGTGACTCGAGTCACACAACATGTAAACCATTTTTG-3′. pLKO.1 vector expressing sh-RNAs targeting EHMT2/G9a (shG9a) were obtained from Prof. Lorenz Poellinger (Cancer Science Institute of Singapore). Specific TRCN clone numbers for shG9a were TRCN0000115667, TRCN0000115670 and TRCN0000416235. All TRCN clones used in this study were verified by sequencing and the information of all the TRCN clines is available on the Genetic Perturbation Platform.

To package the lentivirus, plasmids were co-transfected with the 3rd generation packaging plasmids pMDLg/pRRE (Addgene#12251), pRSV-Rev (Addgene#12253) and pMD2.G (Addgene#12259), which were a kind gift from Dr. Takomi Sanda’s lab (Department of Medicine, Cancer Science Institute of Singapore, Yong Loo Lin School of Medicine, National University of Singapore), into HEK-293T cells using Fugene6^®^ transfection reagent (Promega, cat#E269A). Transfected HEK-293T cells were cultured in DMEM overnight and then changed to RPMI 1640 medium afterwards. 48 h after transfection the lentiviral particles were collected from the supernatants and concentrated up to 50–80 times by centrifugation using Amicon Ultra-100 (Millipore; #UFC910024). 1 mL concentrated lentivirus containing medium was added to MM cells seeded as 0.5 million/well in 6-well plates at 1:1 ratio. The plate was spun at 2500 rpm by a tabletop centrifuge for 1.5 h at room temperature before moved to the incubator. After incubated at 37 °C with 5% carbon dioxide overnight, the lentivirus containing medium was removed and replaced by fresh RPMI 1640 medium. 72 h post infection, the virus-infected cells were selected in RPMI 1640 medium with 2 μg/mL puromycin. After 72 h selection, virus-infected cells were amplified and collected for detection of gene silencing efficiency by immunoblotting for the respective proteins.

### Cell viability assay and determination of half maximal inhibitory concentrations (IC50)

Cell viability was assessed by CellTiter 96^®^ AQueous One Solution (MTS) Cell Proliferation Assay and CellTiter-Glo^®^ (CTG) Luminescent Cell Viability Assay (Promega, Madison, WI, USA). Cells were seeded in 96-well plates (Nunc) at a density of 60,000 cells per mL, 50 μl per well. On the next day, another 50 μl indicated drugs (concentrations ranging from 0.0001 to 100 μM) were added onto cells per treatment (n = 6) and incubated for 72 h. CellTiter 96^®^ AQueous One Solution Cell Proliferation Assays and CellTiter-Glo^®^ Luminescent Cell Viability Assays were conducted following the instructions for use of product. The absorbance at 490 nm or luminescence at 590 nm generated by the cells in each well was recorded with a microplate reader (Tecan, Maennedorf, Switzerland). Each experiment has triplicates. Mean and standard deviation (SD) were calculated. Dimethylsulphoxide (DMSO) treated cells were used as control. Background luminescence (readout of the well with no cell seeded) was subtracted from each sample before calculating the luminescent ratios. The percentage of cell viability of other wells were normalized to the DMSO control. Sigmoidal dose–response curves were generated by fitting calculated cell viability values at different log concentrations while IC_50_ was calculated using GraphPad Prism (v7.0).

### Proliferation assay

G9a-depleted MM cells with control cell lines infected by lentiviruses expressing shNT were seeded in 96-well plates (Nunc) at a density of 20,000 cells per mL, 2000 cells per well on day 0. CellTiter-Glo^®^ Luminescent Cell Viability Assay was conducted on the seeded cells (n = 6) on day 0, day 1, day 2, day 3 and day 4. The day 0 luminescent readout of each cell lines was measured as 1 and following cell viability was normalized accordingly, which was marked as fold change in cell viability and used to measure proliferation for the cell lines.

### Western blot analysis

Whole-cell lysates were prepared from snap frozen cell pellets using RIPA lysis buffer with Complete Mini Protease inhibitors and Phosphatase inhibitors (Roche). Nuclear and Cytoplasmic extracts were prepared from cell pellet or tissue sample using NE-PER™ Nuclear and Cytoplasmic Extraction Kit (Thermo Scientific). Equal amount of the whole cell lysates or nuclear/cytosolic extracts were loaded and separated by SDS–PAGE and transferred to PVDF membrane (Bio-Rad, Hercules, CA, USA), followed by immunoblotting analysis with antibodies to EHMT2/G9a (abcam, ab185050), Di-Methyl-Histone H3 (cell signaling, #4658), Histone H3K9me1 (santa cruz, #39681), Histone H3 (cell signaling, #4499), Tri-Methyl-Histone H3 (cell signaling, #4909), β-actin (Sigma, A2228), phospho-RelB (cell signaling, #5025), RelB (cell signaling, #10544), Bim (cell signaling, #5392), NF-κB2 (cell signaling, #3017), HDAC4 (cell signaling, #5392), HRP-conjugated anti-Rabbit (Cell Signaling #7074), and HRP-conjugated anti-Mouse (Cell Signaling #7076). All primary antibodies were used at 1:2000 dilution and secondary antibody used at 1:10,000 dilution. The blots were developed using ChemiDoc™ MP Imaging System (Bio-rad).

### Quantitative real time PCR (qRT-PCR)

Total RNA of treated cells was extracted from cell pellet using the Qiagen (Venlo, Limburg, Netherlands) RNeasy mini kit and 1 μg RNA was used for cDNA synthesis in 20 μl reactions using iScript Reverse Transcription Supermix (Bio-rad) according to manufacturers’ instructions. Real Time PCR was performed on Biosystems Prism 7500 sequence detection system for triplicate of each cDNA sample (2.5 μl) by SYBR green method. Primers used were as follows: EHMT2 (forward primer, 5′-CTGTCAGAGGAGTTAGGTTCTGC-3′; reverse primer, 5′-CTTGCTGTCGGAGTCCACG-3′), RelB (forward primer, 5′-CAGCCTCGTGGGGAAAGAC-3′; reverse primer, 5′-GCCCAGGTTGTTAAAACTGTGC-3′) BCL2L11/Bim (forward primer, 5′-TCAACACAAACCCCAAGTCC -3′; reverse primer, 5′- TAACCATTCGTGGGTGG TCT-3′), BMF (forward primer, 5′-GAGGTACAGATTGCCCGAAA-3′; reverse primer, 5′-CCCCGTTCCTGTTCTCTTCT-3′) and GAPDH (forward primer, 5′-AGCCACATCGCTCAGACAC-3′; reverse primer, 5′-GCCCAATACGACCAAATCC-3′) HPRT (forward primer, 5′-CCTGGCGTCGTGATTAGTGAT-3′; reverse primer, 5′-AGACGTTCAGTCCTGTCCATAA-3′). The level of gene expression was determined using the ddCT method, normalizing against endogenous control GAPDH or HPRT. Mean ± SD of the triplicate PCR reactions were shown in the results.

### Methylcellulose colony formation assay

Cells were seeded in duplicate at a density of 1000 cells per mL Methylcellulose media (H4230, MethoCult^®^, StemCell Technologies) which contains 1% methylcellulose, 30% fetal bovine serum (FBS), 100 U/mL penicillin and 100 mg/mL streptomycin, and 1% bovine serum, in 35 mm dishes (Corning) as per manufacturer’s recommendations. Dishes were incubated in a humidified atmosphere at 37 °C with 5% carbon dioxide for 14 days and the colonies were counted under an Olympus IX71 inverted microscope. Images were acquired using a digital camera DP71 and software DP Controller.

### In vivo tumor model and tumor growth

1,000,000 KMS12BM cells transfected with lentiviruses expressing shNT or shG9a were injected subcutaneously into the flanks of NOD-scid IL2Rgnull-3/GM/SF, NSG-SGM3 (NSGS) mice. Tumorigenesis measurement started on day 10 until the tumor volume of the 2 out of 3 mice reached the humane endpoint of approximately 1000 mm3; tumor volume was calculated as π/6 × length^2^ × width. Tumor samples for western blotting analysis were snap frozen in liquid nitrogen. All animal studies were done in accordance with protocols approved by the National University of Singapore Institutional Animal Care & Use Committee (IACUC).

### RNA-Seq

RNA was extracted using the RNAeasy Mini Kit as per the manufacturer’s protocol (Qiagen). Sample quality was assessed using NanoDrop™ 2000 Spectrophotometers. RNA sequencing was done on Hiseq 4000, PE100bp sequencing platform (BGI, China) using Truseq RNA library (30Million clean reads), and quality was evaluated with the FastQC [46]. To analyze differentially expressed genes after G9a knockdown, the datasets were aligned using STAR (version 2.5.3a) followed by Rsubread (version 1.34.1) and DESeq 2 (version 1.18.1). Data were analyzed based on shG9a independently as well as cohesively, compared to shNT. Genes are considered differentially expressed when the log2foldchange is smaller than -1 and larger than 1, and the p-values are less than 0.05. In pre-ranked gene set enrichment analysis (GSEA), results from DESeq 2 were first ranked based on an algorithm featured by Mark Ziemann (http://genomespot.blogspot.com/2014/09/data-analysis-step-8-pathway-analysis.html). All ‘NA’ results for log2foldchange and p-values were set to 0 and 1 respectively prior to ranking. Non-canonical NF-κB genesets were downloaded from MSigDB [47] with the keywords “NIK OR nfkb2 OR relb”. Ranked genes were then evaluated in the “Pre-ranked GSEA” module featured in GenePattern [48]. The human genome (GRCh37.p13 Release 19) sequences and annotations from Gencode [49] were used as reference.

### Analysis of CoMMpass RNA-seq dataset

To elucidate the molecular mechanism of G9a conferred NF-κB signaling activation in MM patients, we analyzed a publicly available RNA-seq dataset of CoMMpass (relating Clinical Outcomes in MM to Personal Assessment of Genetic Profile) study, a flagship Personalized Medicine Initiative study of Multiple Myeloma Research Foundation (MMRF). We downloaded TPM-normalized transcriptome and clinical information data (IA10c version) from MMRF’s website (https://research.themmrf.org) and utilized them for survival as well as pathway signature analyses. The transcriptome data went through further preprocessing steps. We first averaged multiple assay profiles of a patient and then took a log_2_-transformation as *J *=* log*_*2*_*(T *+ *0.25) *−* log*_*2*_*(0.25)* where *T* is the TPM value. This can stabilize the variance fluctuation for low level expression transcripts. All subsequent transcriptome analyses used *J* values.

To perform survival analysis, we transformed the survival data units from days to months as *OS *=* oscdy*/*30.44*, *PFS *=* pfscdy*/*30.44* and performed Cox proportional hazard regression analysis using the log_2_-transformed G9a expression values and the month-based survival data. Also, Kaplan–Meier curves were produced by comparing respective survival probabilities of patient groups built after stratifying patients into top 25%, middle 50%, bottom 25% groups based on G9a expression or non-canonical NF-κB signaling signature indices estimated below.

To link G9a expression and non-canonical NF-κB signaling, we compiled two lists of non-canonical NF-κB signaling signatures from two publications [50, 51]. From the first publication, the following 11 genes were obtained: BIRC2/ENSG00000110330, BIRC3/ENSG00000023445, CHUK/ENSG00000213341, MAP3K14/ENSG00000006062, NFKB2/ENSG00000077150, NLRP12/ENSG00000142405, OTUD7B/ENSG00000264522, RELB/ENSG00000104856, TBK1/ENSG00000183735, TRAF2/ENSG00000127191, TRAF3/ENSG00000131323. From the second publication, the following 9 genes were obtained: BIRC2/ENSG00000110330, BIRC3/ENSG00000023445, CD40/ENSG00000101017, CYLD/ENSG00000083799, LTBR/ENSG00000111321, MAP3K14/ENSG00000006062, TNFRSF13B/ENSG00000240505, TRAF2/ENSG00000127191, TRAF3/ENSG00000131323. Then, non-canonical NF-κB signature indices were estimated by first normalizing each of a gene’s expression values with the median of that gene’s overall expression values and then summing up all member genes’ median-normalized expression profile for each sample.

## Results

### G9a is highly expressed in multiple myeloma (MM) cells

**Fig. 1:**
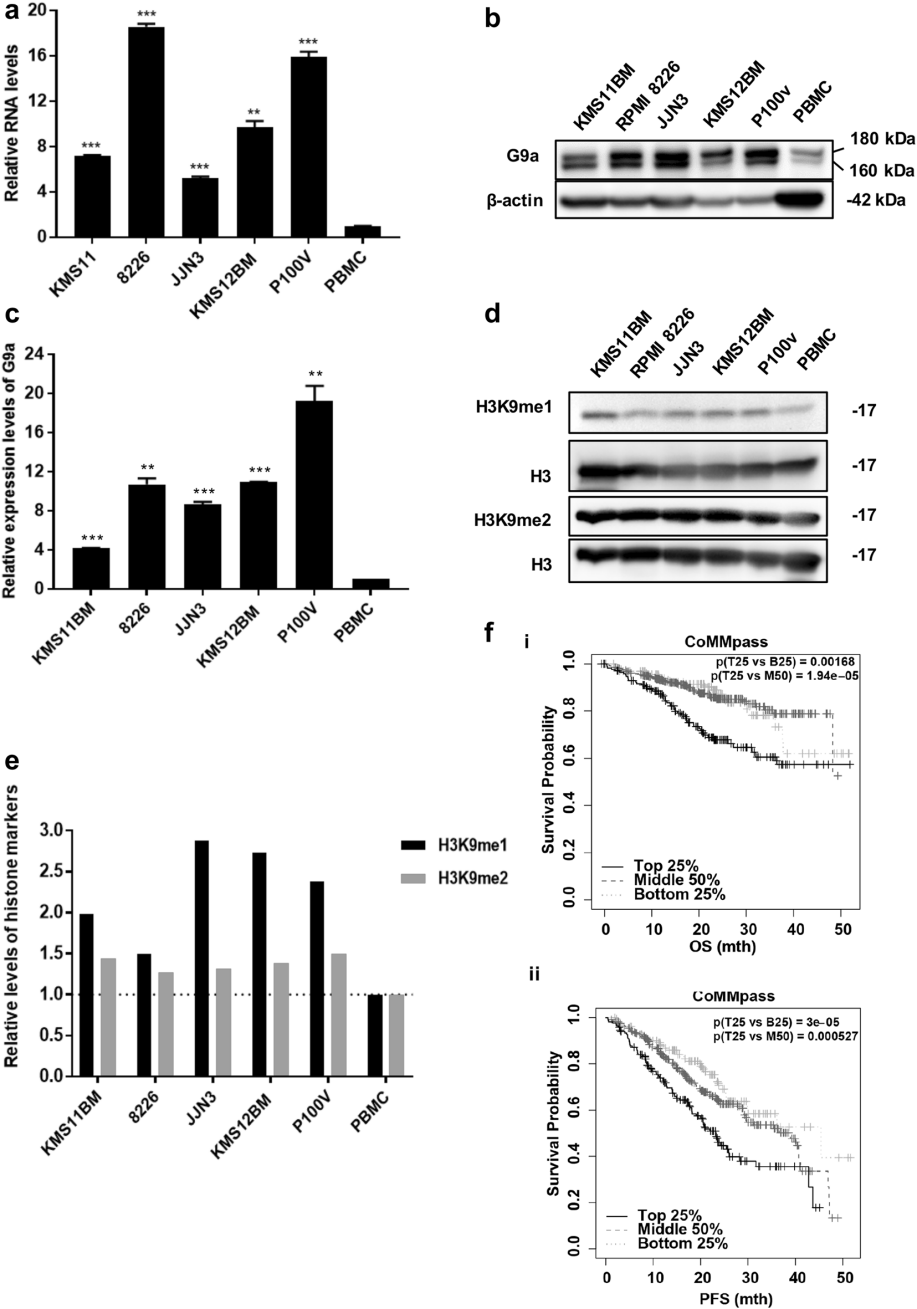
G9a has high expression in multiple myeloma (MM) and regulates H3K9me2. **a** mRNA expression of EHMT2 in multiple myeloma cell lines and peripheral blood mononuclear cell (PBMC). (n = 3; mean ± SD; **p < 0.01 and ***p < 0.001). **b** Immunoblots show G9a in MM cell lines and PBMC. **c** Quantification of G9a band intensity relative to β-actin. (n = 2; mean ± SD; **P < 0.01 and ***p < 0.001). **d** Immunoblots show H3K9me2 and H3K9me1 in mm cell lines and PBMC. **e** Quantification of H3K9me1 and H3K9me2 bands intensity relative to total H3. **f** G9a expression and patient (ii) overall survival with p = 1.25 × 10^−6^ and (i) progression-free survival with p = 2.94 × 10^−7^ in CoMMpass data

While large-scale epigenetic studies have revealed that EHMT2/G9a copy number amplification is frequently observed in MM, the role of G9a in MM, through epigenetic deregulation has been widely observed in MM patients [19, 52]. To examine G9a expression in MM, RNA levels of EHMT2/G9a were analysed in several MM cell lines (MMCLs) compared to peripheral blood mononuclear cells (PBMCs) and normal plasma cells, the CD38^+^ PBMCs as control. As observed in Fig. 1a and Additional file 1: Figure S1A, MMCLs had significantly increased expression of both isoforms of G9a compared to PBMC (p < 0.01) and normal plasma cells (p < 0.001). G9a protein expression analysis confirmed that MMCLs overexpress G9a compared to normal cell controls (Fig. 1b, c and Additional file 1: S1B). According to a previous study, those tested MMCLs had both the non-canonical and canonical pathways activated, except for KMS12BM which has predominant canonical pathway activation [53]. As shown in Fig. 1b and Additional file 1: Figure S1B, MMCLs had two clear bands of two G9a isoforms, while the control cells had little G9a expression. H3K9 mono- and dimethylation levels were also higher in MMCLs than in control cells, which was consistent with the fact that G9a mediates mono- and dimethylation of H3K9 (Fig. 1d, e). In fact, the analysis of CoMMpass data showed that MM patients with high G9a level had relatively poor overall survival (OS) and progression-free survival (PFS) when compared to those with low or medium G9a level (Fig. 1f, Additional file 1: Tables S1, S2). Furthermore, this association was irrespective of other defining patient characteristics. As such, G9a may be a potential therapeutic target against MM.

### G9a-dependent inhibitor sensitivity in MM cells

**Fig. 2:**
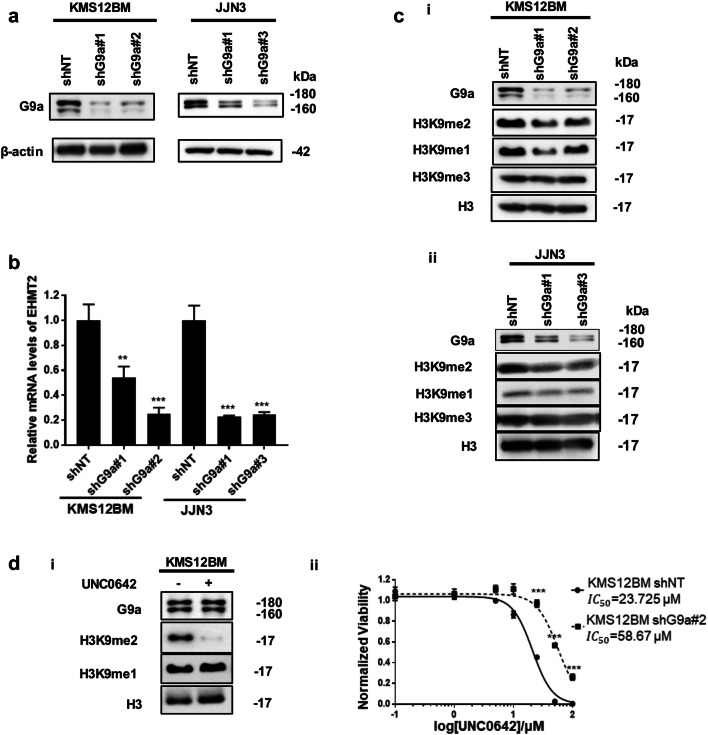
G9a depletion and G9a/GLP inhibitor UNC0642 reduce histone methylation at H3K9me2 and H3K9me1. **a** Immunoblots show G9a and β-actin (loading controls) in indicated MM cell lines infected with lentiviruses expressing Non-targeting (NT) ShRNA (shNT) or sh-RNAs targeting EHMT2 (shG9a). **b** mRNA expression of EHMT2 in indicated MM cell lines infected with lentiviruses expressing shNT or shG9a (n = 3; mean ± SD; Bonferroni corrected one-way ANOVA compared with Non-targeting control; **P < 0.01 and ***p < 0.001). **c** Immunoblots show HEK9me2, H3k9me1 and H3 (loading controls) in (i) KMS12BM and (ii) JJN3 cells infected with lentiviruses expressing shNT or shG9a. **d** (i) Immunoblot analyses shows H3K9me2, H3K9me1 and H3 (loading control) in DMSO (control) or UNC0642 treated KMS12BM cells; 72-h treatment of UNC0642 at $IC_{50}$. (ii) Dose–response curves and $IC_{50}$ of UNC0642 on KMS12BM cells infected with lentiviruses expressing shNT or shG9a. Data are mean ± SD of three independent biological replicates (n = 3; mean ± SD; ***p < 0.001)

In order to evaluate the therapeutic potential of G9a in MM, MMCLs’ sensitivity to the G9a/GLP inhibitor UNC0642 was first evaluated. As shown in Additional file 1: Figures S2Ai and ii, MMCLs exhibited increased sensitivity to UNC0642 compared to normal MSC control cells, however, normal plasma cells also exhibited sensitivity to G9a/GLP inhibition. Loss-of-function studies were performed to determine how much of MMCL sensitivity to G9a/GLP inhibitor UNC0642 as well as observed elevated levels of H3K9 mono- and dimethylation in MMCLs were mediated specifically by G9a. KMS12BM and JJN3 MMCLs were infected with lentiviruses expressing non-targeting shRNA (shNT) or two different shRNAs targeting *EHMT2*/G9a (shG9a). Both shG9a constructs were able to reduce G9a protein and transcript levels compared to shNT in KMS12BM and JJN3 cells (Fig. 2a, b). And as shown in Additional file 1: Figure S3, shRNAs targeting *EHMT2*/G9a was highly specific to reducing *EHMT2* transcription without affecting *EHMT1*/GLP transcription. G9a depletion also corresponded to moderate reduction in both H3K9me2 and H3K9me1 (Fig. 2c). In KMS12BM cells, UNC0642 treatment more potently reduced H3K9me2 levels while G9a protein expression remained unaffected, suggesting that GLP may provide some compensatory effects on H3K9 dimethylation (Fig. 2di). G9a depletion desensitized both KMS12BM and JJN3 to UNC0642 treatment as the IC_50_ of UNC0642 more than doubled in G9a-depleted KMS12BM and JJN3 cells compared to the IC_50_ of UNC0642 of KMS21BM shNT control cells and JJN3 shNT cells (Fig. 2dii and Additional file 1: S2B). Taken together, the results suggest that G9a mediates H3K9 monomethylation and partially mediated H3K9 dimethylation in MM cells and sensitivity to G9a/GLP inhibitor UNC0642.

### G9a contributes to MM cell survival

**Fig. 3:**
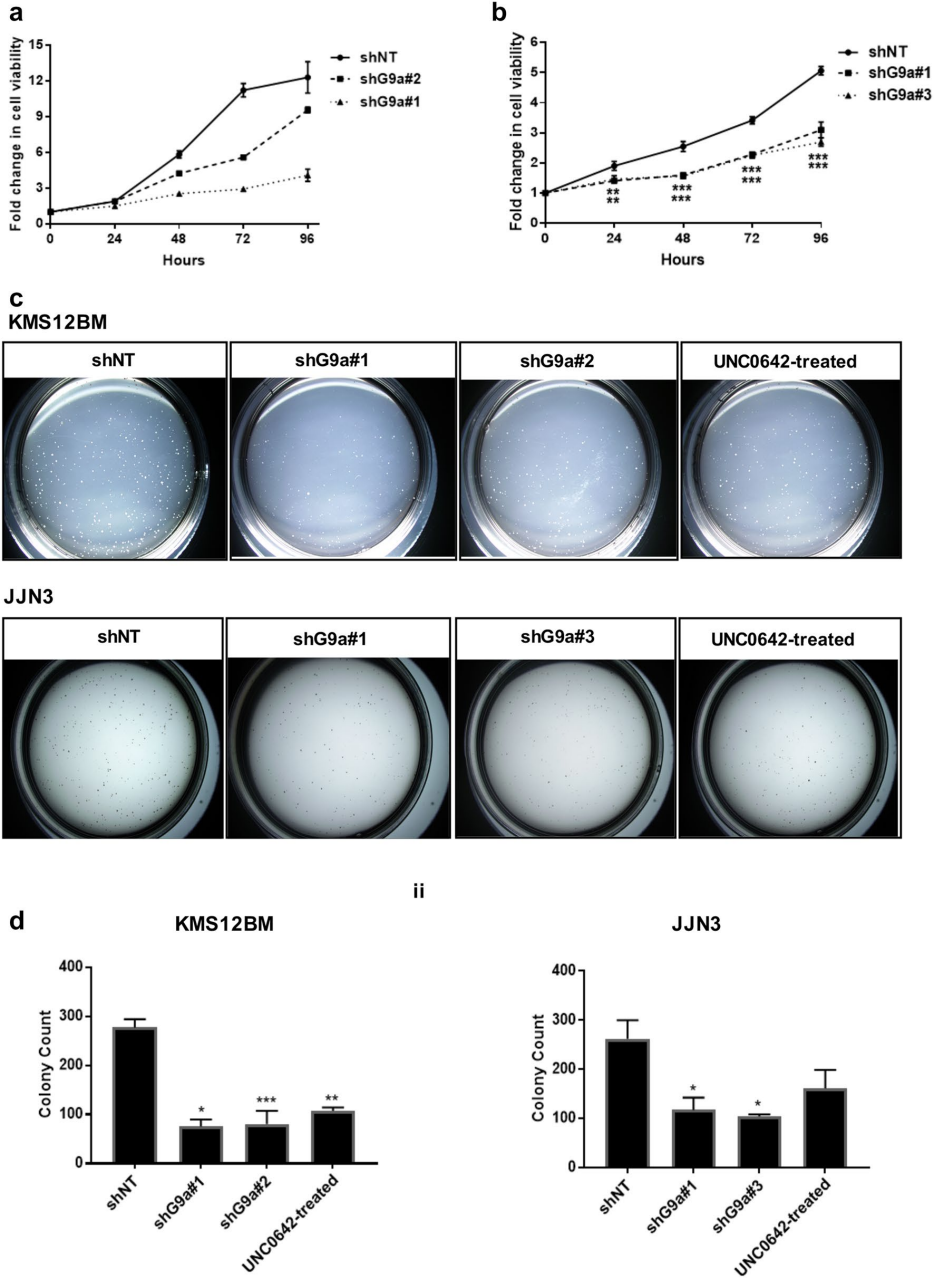
G9a depletion inhibit proliferation of MM cells in vitro. Proliferation assay on G9a-depleted MM cells with control cell lines infected by lentiviruses expressing shNT. KMS12BM (**a**) and JJN3 (**b**). Luminescent cell viability assay was conducted with CellTiter-Glo^®^ at indicated time points. The viability at day0 was measured as 1 and following cell viability was normalized accordingly. Two-tailed Student’s t test, *P < 0.05, **P < 0.01 and ***p < 0.001. **c** KMS12BM and JJN3 cells were infected with lentiviruses expressing shNT or shG9a and 24 h later equal number of cells were seeded into semisolid methyl-cellulose cultures with G9a/GLP inhibitor UNC0642 treated cells as positive control. Colony formation was followed for on day 14 and the images and numbers of colonies were presented as indicated. **d** Quantification of the colony formation assay. On day 14, colonies with size bigger than 100 cells were counted. N = 2, data shown are mean ± S.D. *P < 0.05, **P < 0.01 and ***p < 0.001

**Fig. 4:**
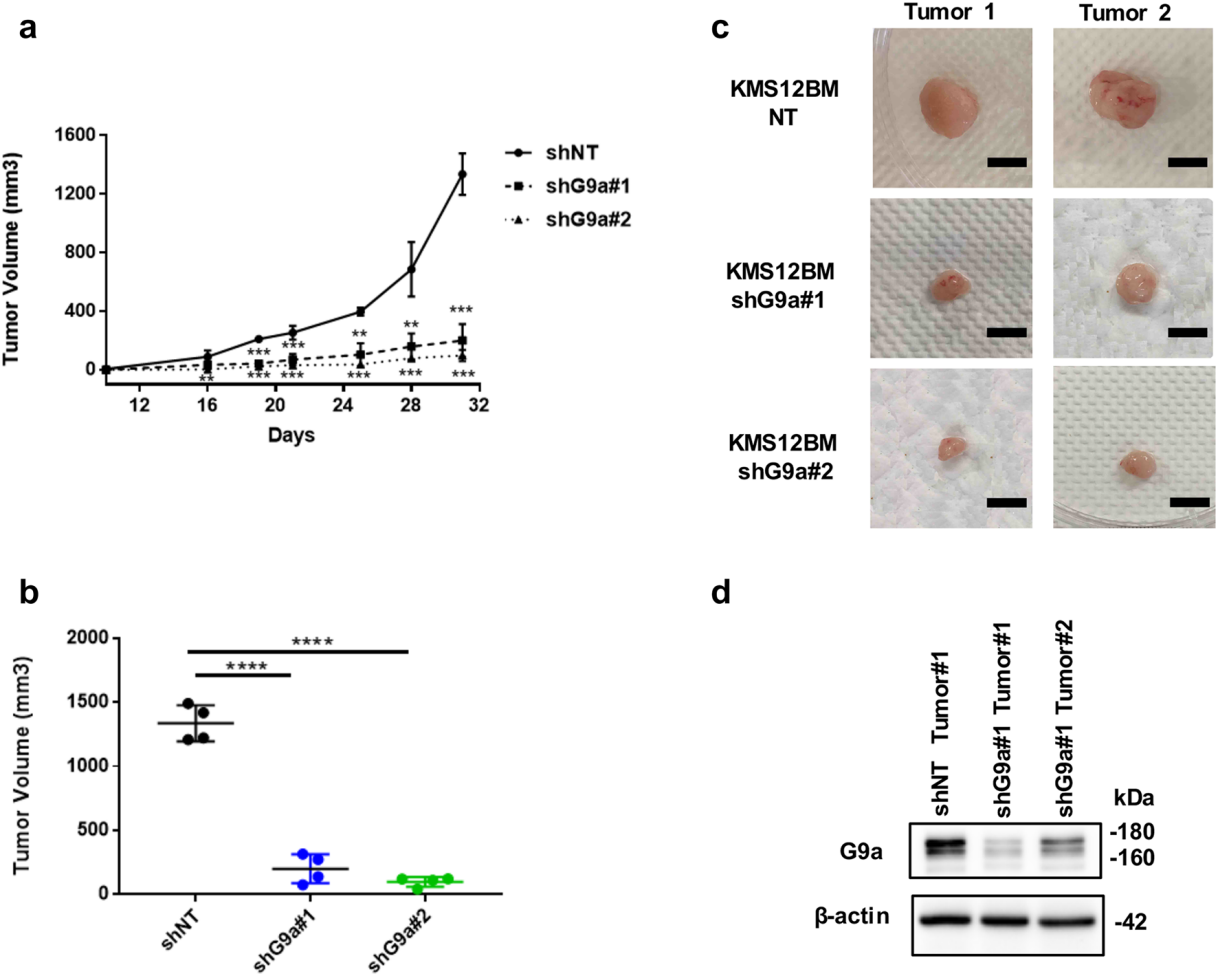
G9a depletion inhibit proliferation of MM cells in vivo. **a** Tumor volumes of KMS12BM xenografts with transfected shNT or shG9a lentivirus over time. (mean ± SD; *p < 0.05, ***p < 0.001 for both comparisons of shNT versus shG9a#1 and shG9a#2, Bonferroni corrected one-way ANOVA). **b** Final individual and mean tumor volumes of KMS12BM xenografts with transfected shNT or shG9a lentivirus at day 31. (Bonferroni corrected one-way ANOVA comparing all groups, ***p < 0.001). **c** Representative tumor images of KMS12BM MM xenografts with transfected shNT or shG9a lentivirus on day31. **d** Immunoblots show G9a expression level in representative KMS12BM xenograft tumors with transfected shNT or shG9a lentivirus

In order to better understand the specific role of G9a in MM pathogenesis as well as the effect of G9a-specific loss in MM, tumor cell viability and clonogenic properties were evaluated in loss-of-function studies. In Fig. 3a, b, depletion of G9a resulted in significantly impaired cell survival in both KMS12BM and JJN3. Moreover, both G9a knockdown by shG9a and G9a/GLP inhibition by UNC0642 treatment significantly reduced the size and number of colonies formed (Fig. 3c, d). This result further confirmed a role for G9a in promoting pro-tumorigenic phenotypes in MMCLs. To further investigate the effect of G9a in MM tumor pathogenesis, MM tumor growth was investigated in vivo following G9a depletion. KMS12BM cells transfected with shG9a or shNT were injected into mice subcutaneously and tumor volumes were measured on indicated days from the tenth day on. G9a depletion significantly decreased tumor volume and impaired tumorigenesis in vivo as compared to the shNT control (Fig. 4a–d). Collectively, these results together suggested that G9a contributes to tumorigenesis, tumor cell viability and tumor progression. As such, these data provide further evidence that G9a may be a valid therapeutic target in the treatment of MM (Table 1).

**Table 1:** Tumor volumes at endpoint

	shNT	shG9a#1	shG9a#2
Tumor 1	1208.86	74.82	40.84
Tumor 2	1491.62	137.61	118.46
Tumor 3	1222.87	314.02	109.66
Tumor 4	1419.61	272.10	120.70

### G9a positively regulates NF-κB pathway in MM

In order to investigate the molecular mechanism by which G9a regulate the growth of MM cells, we analyzed transcriptome-wide changes in KMS12BM cells after G9a depletion via RNA sequencing (RNA-seq) followed by differential gene expression (DE) analysis and pathway analysis using PANTHER pathway database. The pathway analysis of differentially expressed genes identified that ‘Inflammation mediated by chemokine and cytokine signaling pathway (P00031)’ was the most disturbed following G9a depletion (Additional file 1: Figure S4A). Based on that, pathway analysis was performed specifically for genes significantly downregulated following G9a depletion in the MM cells. As shown in Table 2, inflammation pathway ranked top in the downregulated pathways, and the apoptosis signaling pathway ranked second. Both ATF3 and ATF4 were identified as significantly downregulated and have previously been reported to serve a proapoptotic function in MM [54, 55]. Interestingly, in both pathways we noticed that RelB, the main subunit of non-canonical NF-κB pathway, was significantly downregulated by G9a depletion (Table 3).

**Table 2:** Overall downregulated differentially genes from the pathway analysis (PANTHER pathway database)

	Pathway	Entities found	Genes	RNA expression (fold change)	p-values
1	Inflammation mediated by chemokine and cytokine signaling pathway (P00031)	5	ACTA2	0.330	0.0000675
			RELB	0.235	1.27E−11
			C5AR1	0.133	0.00286572
			ITGB7	0.491	0.000876315
			PRKACB	0.485	0.000301808
2	Apoptosis signaling pathway (P00006)	4	RELB	0.235	1.27E−11
			ATF4	0.419	0.00000206
			ATF3	0.352	0.000000508
			JDP2	0.278	0.00000913
3	Integrin signalling pathway (P00034)	3			
4	p53 pathway (P00059)	3			
5	Cholesterol biosynthesis (P00014)	3

**Table 3:** Leading edge subset of GSEA GO_NIK_NF_KAPPAB_SIGNALING

Probe	Rank in gene list	Rank metric score	Running es	Core enrichment	log2FoldChange	p value
RELB	57316	− 10.699	5.94E−04	Yes	− 2.08872	1.27E−11
TNFRSF10B	57177	− 4.84771	− 0.102165	Yes	− 1.16134	1.19E−05
PSMC2	57153	− 4.62525	− 0.149415	Yes	− 1.21042	1.97E−05
BIRC3	56951	− 3.31569	− 0.191371	Yes	− 1.14829	0.000472
NFKB2	56761	− 2.66561	− 0.247862	Yes	− 0.94940976	5.26E−05
UBC	56752	− 2.64281	− 0.273925	Yes	− 0.745182523	0.000295

**Fig. 5:**
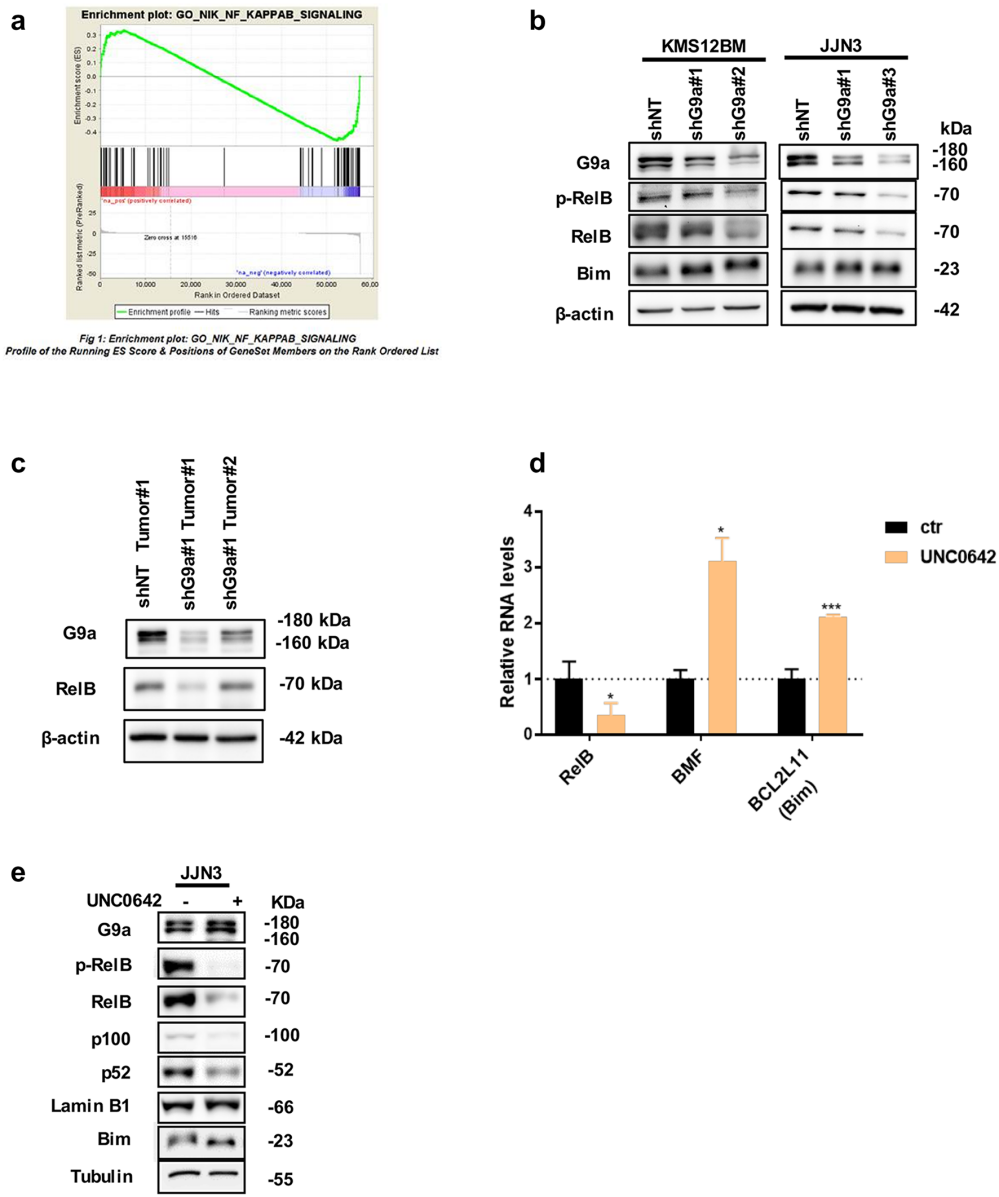
G9a depletion inhibit proliferation of MM cells in vitro. **a** RNA-seq performed on KMS12BM transfected with lentiviruses expressing shNT or shG9a (n = 2). Gene set enrichment analysis plots of negative enrichment (FDR = 0.41) for NFκB pathway when comparing shG9a#1 and #2 versus shNT. NER = -1.0079; FDR q-value = 0.41. NES, normalized enrichment score. **b** Immunoblots show G9a, p-RelB, RelB and proapoptotic factor Bim (β-actin as loading control) in indicated MM cell lines infected with lentiviruses expressing shNT or shG9a. **c** Immunoblots show RelB expression levels in representative KMS12BM xenografts with transfected shNT or shG9a lentivirus. **d** mRNA expression of RelB and proapoptotic factors BMF, BCL2L11 (Bim) in JJN3 cells treated by DMSO control or UNC0642 at $IC_{50}$ for 72 h. (n = 3; mean ± SD; Two-tailed Student’s t test compared with DMSO treated control; *P < 0.05 and ***p < 0.001). **e** Whole cell lysates from JJN3 cells treated by DMSO control or UNC0642 at $IC_{50}$ for 72 h were analyzed for the indicated proteins by immunoblotting

The NF-κB family of transcription factors drive transcriptional activation of apoptosis regulating and proinflammatory genes such as cytokines and chemokines, regulating overall immune response [56]. In MM, activation of both canonical and non-canonical NF-κB pathways are prevalent, which creates inflammatory microenvironment for tumor growth and have been found to be associated with disease progression [11, 53, 57]. Moreover, previous studies have shown that transcriptional repression by NF-kB can also promote survival in MM cells [42]. To further investigate how loss of G9a affects both canonical and non-canonical NF-κB pathway, we evaluated an NF-κB gene set, GO_NIK_NF_KAPPAB_SIGNALING obtained from Molecular Signatures Database, using gene set enrichment analysis (GSEA). We observed that a number of genes within the NF-κB pathway gene set were repressed following loss of G9a (Fig. 5a). Importantly, among the leading-edge subset of GSEA, the genes of non-canonical NF-κB pathway such as RelB and NFKB2 were found to be in the repressed gene group contributing substantially to the core enrichment (Table 3). When we compared the expression of G9a and two non-canonical NF-κB signature indices, we found very tight correlation among them in CoMMpass data (Additional file 1: Figure S4B). As expected, the two NF-κB signature indices exhibited very high correlation (*r *=0.9*, p* < 2.2 × 10^−16^). Interestingly, the G9a expression and two non-canonical NF-κB signature indices showed very high correlation as well (*r *=0.5 and 0.45, both *p* < 2.2 × 10^−16^). Collectively, these results indicated the regulation of NF-κB pathway by the epigenetic modifier G9a in MM and that depletion of G9a reduced the activity of NF-κB pathway, especially the non-canonical NF-κB pathway.

In previous studies, RelB-p52 was reported to function as a transcriptional repressor for Bim and BMF genes and the constitutive phosphorylation of RelB correlated with RelB-dependent survival of MM [42]. Thus, protein expression of RelB, p-Relb and the pro-apoptotic factor Bim was interrogated in two G9a depleted MMCLs. Notable decrease of p-RelB and RelB were observed in G9a depleted MM cells with correspondingly moderate increases in Bim expression (Fig. 5b; Additional file 1: Figure S4C). We next checked the expression level of RelB in tumor samples of previous xenografts finding that the RelB seemed to positively correlate with the G9a levels and tumor volume (Figs. 4c, 5c). In line with the phenotypes observed following G9a knockdown, UNC0642 treatment also reduced the transcription of RelB and elevated the expression of the proapoptotic proteins BMF and Bim (Fig. 5d). Furthermore, clear decreases of NFKB2/p100/p52 in JJN3 cells can be detected after 72-h UNC0642 treatment (Fig. 5e).

**Fig. 6:**
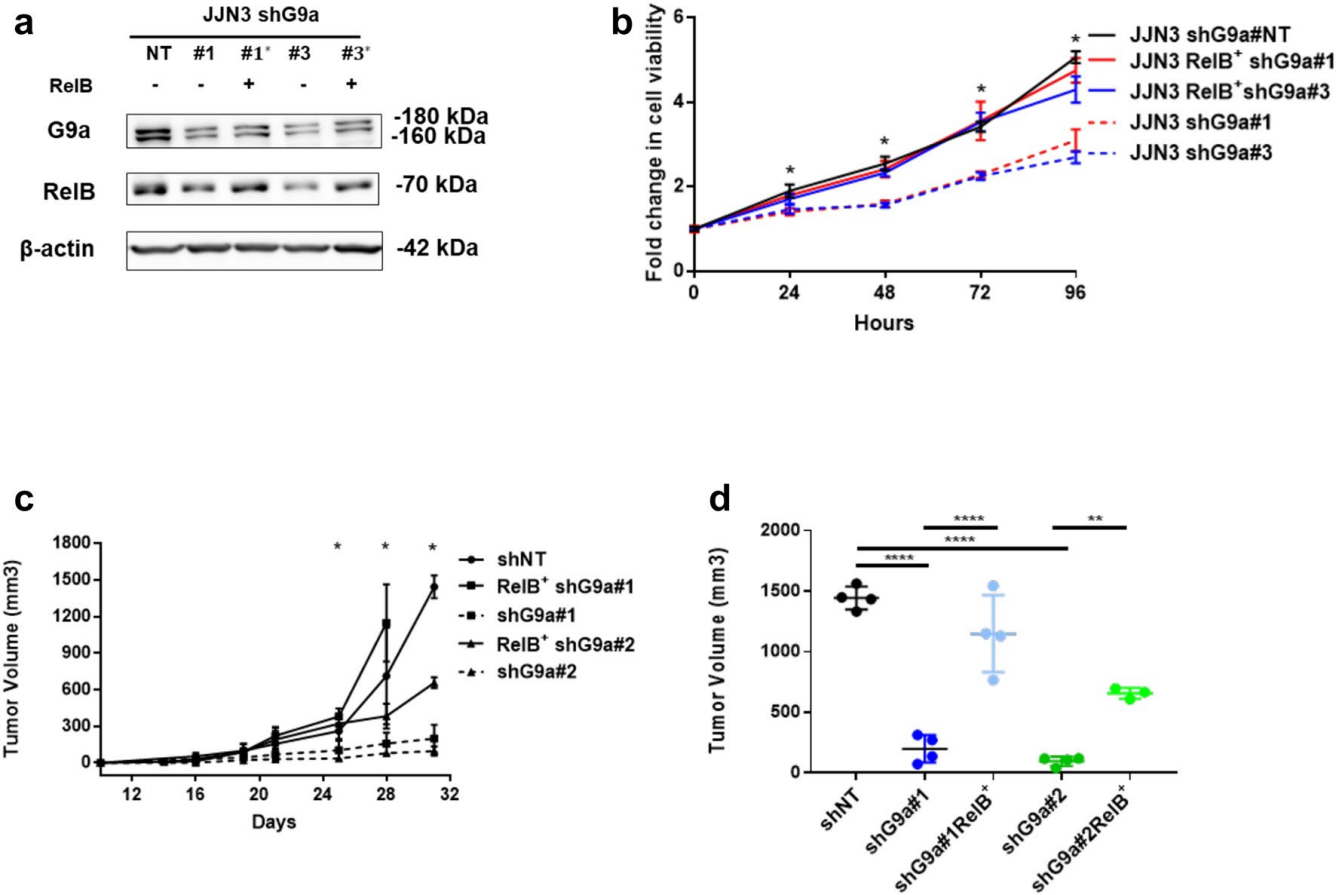
Rescue RelB in G9a depleted MM cells eliminates inhibition of proliferation. **a** Immunoblots show expression levels of G9a and RelB in KMS12BM transfected with lentiviruses expressing shNT or shG9a and RelB recovered G9a depleted KMS12BM cells after long term culture. **b** Proliferation assay on RelB recovered G9a-depleted JJN3 cells with control JJN3 cells infected by lentiviruses expressing shNT. Luminescent cell viability assay was conducted with CellTiter-Glo^®^ at indicated time points. The viability at day0 was measured as 1 and following cell viability was normalized accordingly. Two-tailed Student’s t test, *p < 0.05, for comparisons of shNT, $RelB^{ + }$ shG9a#1 and $RelB^{ + }$ shG9a#2 versus shG9a. **c** Tumor volumes over time of KMS12BM xenografts generated with shNT transfected or shG9a transfected/RelB-recovered KMS12BM cells. (n = 3, mean ± SD; *p < 0.05, **p < 0.01, ***p < 0.001 for comparisons of shNT, $RelB^{ + }$ shG9a#1 and $RelB^{ + }$ shG9a#2 versus shG9a, Bonferroni corrected one-way ANOVA). **d** Final individual and mean tumor volumes of KMS12BM xenografts generated with shNT transfected or shG9a transfected/RelB-recovered KMS12BM cells at day 31. (Bonferroni corrected one-way ANOVA comparing all groups, **p < 0.01, ****p < 0.0001)

In order to investigate the role of RelB in G9a-mediated pro-tumorigenic phenotypes in MM cells, cell proliferation was interrogated in RelB^+^ shG9a MM cells in which the down-regulated RelB following G9a depletion was rescued (Fig. 6a). As shown in Fig. 6b, the proliferation of RelB^+^ shG9a MM cells was completely rescued by upregulated expression of RelB compared to shNT MM cells and significantly differed from the shG9a MM cells that had lower RelB expression (Fig. 6b). Furthermore, overexpression of RelB in G9a-depleted MM cells rescued tumor growth rate in vivo in MM xenograft mouse models (Fig. 6c, d). Thus, in vivo data confirm the interplay between G9a and RelB observed in vitro. These data provide evidence that G9a can drive an activated NF-κB pathway in MM primarily through positive regulation of RelB/p52. Interestingly, previous studies of G9a have been primarily focused on transcriptional repression roles of G9a through histone modification H3K9me2/1. In the case of MM, G9a may serve as a positive driver of NF-κB in order to mediate a pro-survival gene program in MM cells.

## Discussion

Normal cells undergo numerous biological reprogramming processes in order to transform into cancer cells [58]. This reprogramming is not limited to genomic alterations, but also includes critical changes to the epigenome. Epigenetic changes such as DNA methylation, alterations in noncoding RNA profiles and histone modifications are crucial factors in development, senescence and memory formation [59–61]. Therefore, epigenetic disorder is thought to be impactful on cellular transformation and an important causative factor in several diseases, including the evolution of cancer [62, 63]. For those cancer types with heterogenous, non-dominant genomic alterations or the lack of effectively targetable driving oncogenes, targeting epigenetic modifiers may be an attractive non-canonical therapeutic strategy.

MM is an incurable hematological malignancy, which shows great heterogeneity in both epigenetic and genetic aberrations. Consistent with the finding that EHMT2/G9a copy number amplifications are frequently observed in MM patients in a previous study on MM epigenetic-related genomic alterations [19], we demonstrated that G9a expression was highly upregulated in several genetically diverse MMCLs. G9a is a key epigenetic modifier that mediates the histone modification H3K9me2, which is required for transcriptional repression in euchromatin. The role of G9a up-regulation in MM cells was confirmed by the observation of higher level of H3K9me2/1 in MMCLs compared to normal plasma cells. This evidence of G9a activity in MM is notable given that multiple studies have identified diverse molecular mechanisms by which G9a can contribute to carcinogenesis, tumor pathogenesis and poor survival outcomes in cancer patients [31]. For instance, several studies demonstrated that G9a mediated methylation can promote invasion and metastasis in a number of cancers [33, 36, 64, 65]. G9a can also repress autophagy in cancer cells, the reversal of which can induce autophagic cell death in cancers [34, 66]. Another well described mechanism by which G9a contributes to cancer biology is through both transcriptional repression of genes negatively regulated by hypoxia as well as activating methylation of pro-tumorigenic hypoxic factors, such as Pontin [30, 67, 68]. G9a loss-of-function analysis in this study, by both shRNA and G9a inhibitor, confirmed that G9a mediates H3K9me2/1 in MM cells. More importantly, depletion or inhibition of G9a impairs MM cell growth and viability, both in vitro and in vivo. Furthermore, this regulation appears to be linked directly to G9a expression levels as MM tumor sizes were positively correlated to expression levels of G9a. Collectively, these data suggest that G9a may be an attractive therapeutic target for treating MM. The specificity of G9a expression compared to normal plasma cells as well as the minimal effects of G9a inhibition on normal lymphocyte development further emphasize the attractiveness of targeting G9a in multiple myeloma.

While targeting G9a may be an effective approach to treating multiple myeloma, a number of hurdles remain in the translation of G9a inhibition into the clinic. G9a depletion desensitized some MM cells to the G9a/GLP inhibitor UNC0642, suggesting that the observed H3K9 methylation in MM is driven primarily by G9a. As such, elevated levels of G9a may be an appropriate biomarker for therapeutic intervention of MM by specific G9a inhibitors. However, most G9a inhibitors do not have the necessary PK properties to allow for in vivo applications. The most suitable of these, UNC0642, also strongly inhibits other methyltransferases, including GLP and EZH2. There is evidence that both GLP and EZH2 play key roles in B cell development, with G9a/GLP inhibitors capable of preventing HSC differentiation and EZH2 being critical to germinal center formation [27, 69]. The potential toxicity is evident in the study where UNC0642 exhibited some toxicity against normal plasma cells, but not in MSCs which do not have evidence of a role of G9a, GLP or EZH2 in cell survival. As such, the toxicity of G9a/GLP inhibitors to normal hematopoietic stem cells and plasma cells needs to be considered and properly managed prior to clinical application. There is evidence in other cancers that combination therapy may improve efficacy and safety of G9a-based therapy [70]. A well-designed and optimized drug combinations of G9a inhibitors help to increase the efficacy and as a result, reduce the toxicity as lower dosage of a G9a inhibitor will be needed compared to the dosage when a G9a inhibitor is used as a single drug [45, 71].

Previous studies have found that activating mutations of NF-κB pathway have been frequently observed in MM with nearly 50% of MMCLs and most primary MM samples observed to have increased levels of NF-κB activity [41]. Given the importance of NF-κB pathway in MM pathogenesis, the positive regulation of NF-κB signaling by G9a in MM may have profound significance on understanding and treating MM [72]. Specifically, work here shows that G9a can positively regulate expression of RelB, which together with p52 is the main member of non-canonical NF-κB pathway. Reversal of G9a regulation of RelB through either G9a silencing or pharmacological inhibition can promote a pro-apoptotic signal as evidenced by the induction of proapoptotic genes *BCL2L11 (Bim)* and *BMF.* It is worth noting that the KMS12BM cell line has mainly the activation in canonical NF-κB pathway with invisible NIK level and only basal level of p52, while JJN3 has both canonical and non-canonical NF-κB pathway activated [42, 53, 73]. Thus, targeting the NF-κB pathway in MM through G9a may be effective across a diverse range of MM patients. It has also been reported that the basal RelB-p52 activity was essential for MM survival, even when NIK stabilization was absent [11, 42]. Activated RelB was much more frequently observed than the activation of non-canonical NF-κB pathway in primary MM patient sample [11], both suggesting that there are non-canonical mechanisms to RelB activation other than NIK-based non-canonical NF-κB pathway. In this study, the downregulation of RelB and inhibited survival by G9a depletion was clearly observed in not only JJN3 cells but also the KMS12BM cells, suggesting that this non-canonical NF-κB pathway-independent RelB activation can be regulated by G9a. While there is evidence in normal immune response that RelB and G9a can interact, the mechanism by which G9a regulates RelB expression in MM remains unknown and warrants further study [74, 75]. Unlike traditional transcriptional repression via G9a histone methyltransferase function or G9a-mediated DNA methylation recruiting DNMTs, G9a may positively regulate the expression of RelB through co-activating with other transcriptional activators where G9a functions as a scaffold protein. In gastric cancer studies, G9a was shown to recruit p300 and GR, promoting gastric cancer metastasis by upregulating ITGB3 in a SET-domain independent manner [64] In future studies, interrogation of G9a binding to the RelB promoter region as well as identification of co-transcription factors of G9a may provide greater insight into G9a regulation of RelB.

The positive regulation of RelB and NF-κB pathway in MM makes G9a an attractive therapeutic target for developing novel MM therapies. Previous studies have suggested that there is a common RelB-addiction in MM survival [42]. RelB was found to be essential for MM survival, regardless of the oncogenic events such as NIK activation. Furthermore, inhibition of RelB in several genetically diverse MMCLs, including Bortezomib-resistant MM cells, induced transcription and expression of proapoptotic genes *Bim* and *BMF*, and eventually the cell death [42]. NF-κB pathway plays a role in multiple important biological processes such as inflammation, transformation, cell growth, angiogenesis, metastasis, drug resistance in cancer [76]. Activation of NF-κB transcription factors was found to be critical in the survival and proliferation of MM, with MM growth being highly dependent on the microenvironment containing cytokines and chemokines induced by NF-κB pathway [77]. While there is substantial evidence that targeting NF-κB pathway is an effective therapeutic strategy, there continue to be challenges towards increasing the armamentarium that can target NF-κB pathway in MM. One of the most commonly used inhibitors of NF-κB pathway is bortezomib, a proteasome inhibitor developed for MM treatment based on the proteasome machinery which can inhibit NF-κB pathway activation. Resistance to bortezomib, however, is inevitable and frequently observed among MM patients with activated NF-κB pathway [78]. The bortezomib-resistant NF-κB activity regulated by proteasome inhibitor-resistant (PIR) pathway has been documented, suggesting that there may be unknown proteasome-independent NF-κB regulations which maintains the basal RelB activity contributing to initial and/or gained bortezomib resistance in clinic [79]. Further studies will be done to elucidate G9a-based combinations as a therapeutic option for relapsed/refractory MM following bortezomib-based treatment. Another challenge in targeting RelB or the NF-κB pathway in MM treatment is the overcoming toxic side effects of directly targeting NF-κB due to the pervasive role of NF-κB in normal biology. Collectively, this study reveals that G9a is a key mediator of MM survival and growth by positively regulating RelB. Inhibition of G9a can be a promising approach to selectively block NF-κB pathway in MM, reversing RelB-mediated transcriptional repression of proapoptotic genes, such as *Bim* and *BMF*. The increased expression of G9a and effect of G9a loss-of-function on MM cells makes G9a an attractive target for inhibiting NF-κB-related processes in MM. While G9a-based therapy will likely require combination based therapy with other MM drugs, targeting G9a to selectively inhibit RelB or NF-κB pathway only in MM cells may come with less toxic adverse effects than current broader therapeutic approaches. Therapeutically targeting pro-tumorigenic epigenetic modifiers, such as G9a, may also serve as an effective method for treating genetically diverse MM subtypes and provide additional options for a currently incurable disease.

## Conclusions

In this study, we found that G9a expression was highly upregulated in several genetically diverse MMCLs and G9a played a role in regulation of proliferation and tumorigenesis in MM. G9a depletion and inhibition resulted in impaired MM cell growth and viability both in vitro and in vivo and G9a sensitive the MM cells to G9a/GLP inhibitor UNC0642 including the bortezomib-resistant RPMI 8226 cells (P100v). Thus, elevated levels of G9a may be an appropriate biomarker for therapeutic intervention of MM by G9a inhibitors especially for the MM patients who are refractory or developed resistance to bortezomib. After that, we further identified that one mechanism by which G9a mediates its pro-tumorigenic effects in MM is through the positive regulation of the NF-κB pathway, in particular RelB-mediated signaling. This makes G9a an attractive target as epigenetic modifiers for non-canonical therapeutic strategy.

## Supplementary information

**Additional file 1.** Additional Figures S1-S4; Additional Table S1 and Additional Table S2.

## Data Availability

All data analysed during this study are included in this published article and its additional files.

## Abbreviations

MM: Multiple myeloma
PBMCs: Peripheral blood mononuclear cells
OS: Overall survival
HDAC1: Class I histone deacetylases
CNVs: Copy number variations
EHMT2: Euchromatic histone-lysine *N*-methyltransferase 2
H3K9: Histone H3 lysine 9
GLP: G9a/G9a-like-protein
PRC2-EZH2: Polycomb repressive complex 2-enhancer of zeste 2
MMCLs: Multiple myeloma cell lines
shG9a: sh-RNAs targeting EHMT2/G9a
IC50: Half maximal inhibitory concentrations
SD: Standard deviation
DMSO: Dimethylsulphoxide
qRT-PCR: Quantitative real time PCR
NSGS: NSG-SGM3
FBS: Fetal bovine serum
GSEA: Gene set enrichment analysis
MMRF: Myeloma research foundation
DE: Differential gene expression
MSCs: Mesenchymal stromal cells
PFS: Progression-free survival
shNT: Non-targeting shRNA
PIR: Proteasome inhibitor-resistant

## References

[1] Landgren O, Morgan GJ (2014). Biologic frontiers in multiple myeloma: from biomarker identification to clinical practice. Clin Cancer Res.

[2] Reisenbuckler C (2014). Multiple myeloma and diagnostic imaging. Radiol Technol.

[3] Smith BD, Smith GL, Hurria A, Hortobagyi GN, Buchholz TA (2009). Future of cancer incidence in the United States: burdens upon an aging, changing nation. J Clin Oncol.

[4] Vedadi M, Barsyte-Lovejoy D, Liu F, Rival-Gervier S, Allali-Hassani A, Labrie V (2011). A chemical probe selectively inhibits G9a and GLP methyltransferase activity in cells. Nat Chem Biol.

[5] Orlowski RZ (2013). Why proteasome inhibitors cannot ERADicate multiple myeloma. Cancer Cell.

[6] Landgren O, Rajkumar SV (2016). New developments in diagnosis, prognosis, and assessment of response in multiple myeloma. Clin Cancer Res.

[7] Kuehl WM, Bergsagel PL (2002). Multiple myeloma: evolving genetic events and host interactions. Nat Rev Cancer.

[8] Chng WJ, Glebov O, Bergsagel PL, Kuehl WM (2007). Genetic events in the pathogenesis of multiple myeloma. Best Pract Res Clin Haematol.

[9] Chng WJ, Kuehl WM, Bergsagel PL, Fonseca R (2008). Translocation t(4;14) retains prognostic significance even in the setting of high-risk molecular signature. Leukemia.

[10] Kuehl WM, Bergsagel PL (2012). Molecular pathogenesis of multiple myeloma and its premalignant precursor. J Clin Investig.

[11] Cormier F, Monjanel H, Fabre C, Billot K, Sapharikas E, Chereau F (2013). Frequent engagement of RelB activation is critical for cell survival in multiple myeloma. PLoS ONE.

[12] Alzrigat M, Parraga AA, Jernberg-Wiklund H (2018). Epigenetics in multiple myeloma: from mechanisms to therapy. Semin Cancer Biol.

[13] Dimopoulos K, Gimsing P, Gronbaek K (2014). The role of epigenetics in the biology of multiple myeloma. Blood Cancer J.

[14] Chim CS, Liang R, Leung MH, Yip SF, Kwong YL (2006). Aberrant gene promoter methylation marking disease progression in multiple myeloma. Leukemia.

[15] Walker BA, Wardell CP, Chiecchio L, Smith EM, Boyd KD, Neri A (2011). Aberrant global methylation patterns affect the molecular pathogenesis and prognosis of multiple myeloma. Blood.

[16] Harada T, Ohguchi H, Grondin Y, Kikuchi S, Sagawa M, Tai YT (2017). HDAC3 regulates DNMT1 expression in multiple myeloma: therapeutic implications. Leukemia.

[17] Martinez-Garcia E, Popovic R, Min DJ, Sweet SM, Thomas PM, Zamdborg L (2011). The MMSET histone methyl transferase switches global histone methylation and alters gene expression in t(4;14) multiple myeloma cells. Blood.

[18] Pawlyn C, Kaiser MF, Heuck C, Melchor L, Wardell CP, Murison A (2016). The spectrum and clinical impact of epigenetic modifier mutations in myeloma. Clin Cancer Res.

[19] Dupere-Richer D, Licht JD (2017). Epigenetic regulatory mutations and epigenetic therapy for multiple myeloma. Curr Opin Hematol.

[20] Guryanova OA, Shank K, Spitzer B, Luciani L, Koche RP, Garrett-Bakelman FE (2016). DNMT3A mutations promote anthracycline resistance in acute myeloid leukemia via impaired nucleosome remodeling. Nat Med.

[21] Mithraprabhu S, Kalff A, Chow A, Khong T, Spencer A (2014). Dysregulated Class I histone deacetylases are indicators of poor prognosis in multiple myeloma. Epigenetics..

[22] Laubach JP, Moreau P, San-Miguel JF, Richardson PG (2015). Panobinostat for the treatment of multiple myeloma. Clin Cancer Res.

[23] Maes K, Menu E, Van Valckenborgh E, Van Riet I, Vanderkerken K, De Bruyne E (2013). Epigenetic modulating agents as a new therapeutic approach in multiple myeloma. Cancers..

[24] Fratta E, Montico B, Rizzo A, Colizzi F, Sigalotti L, Dolcetti R (2016). Epimutational profile of hematologic malignancies as attractive target for new epigenetic therapies. Oncotarget..

[25] Walker BA, Boyle EM, Wardell CP, Murison A, Begum DB, Dahir NM (2015). Mutational spectrum, copy number changes, and outcome: results of a sequencing study of patients with newly diagnosed myeloma. J Clin Oncol..

[26] Tachibana M, Matsumura Y, Fukuda M, Kimura H, Shinkai Y (2008). G9a/GLP complexes independently mediate H3K9 and DNA methylation to silence transcription. EMBO J.

[27] Chen X, Skutt-Kakaria K, Davison J, Ou YL, Choi E, Malik P (2012). G9a/GLP-dependent histone H3K9me2 patterning during human hematopoietic stem cell lineage commitment. Genes Dev.

[28] Esteve PO, Chin HG, Smallwood A, Feehery GR, Gangisetty O, Karpf AR (2006). Direct interaction between DNMT1 and G9a coordinates DNA and histone methylation during replication. Genes Dev.

[29] Thomas LR, Miyashita H, Cobb RM, Pierce S, Tachibana M, Hobeika E (2008). Functional analysis of histone methyltransferase G9a in B and T lymphocytes. J Immunol.

[30] Lee JS, Kim Y, Bhin J, Shin HJ, Nam HJ, Lee SH (2011). Hypoxia-induced methylation of a pontin chromatin remodeling factor. Proc Natl Acad Sci USA.

[31] Casciello F, Windloch K, Gannon F, Lee JS (2015). Functional role of G9a histone methyltransferase in cancer. Front Immunol.

[32] Huang J, Dorsey J, Chuikov S, Perez-Burgos L, Zhang X, Jenuwein T (2010). G9a and Glp methylate lysine 373 in the tumor suppressor p53. J Biol Chem.

[33] Chen MW, Hua KT, Kao HJ, Chi CC, Wei LH, Johansson G (2010). H3K9 histone methyltransferase G9a promotes lung cancer invasion and metastasis by silencing the cell adhesion molecule Ep-CAM. Cancer Res.

[34] Li KC, Hua KT, Lin YS, Su CY, Ko JY, Hsiao M (2014). Inhibition of G9a induces DUSP4-dependent autophagic cell death in head and neck squamous cell carcinoma. Mol Cancer..

[35] Wei L, Chiu DK, Tsang FH, Law DC, Cheng CL, Au SL (2017). Histone methyltransferase G9a promotes liver cancer development by epigenetic silencing of tumor suppressor gene RARRES3. J Hepatol.

[36] Liu XR, Zhou LH, Hu JX, Liu LM, Wan HP, Zhang XQ (2018). UNC0638, a G9a inhibitor, suppresses epithelialmesenchymal transitionmediated cellular migration and invasion in triple negative breast cancer. Mol Med Rep.

[37] Guo AS, Huang YQ, Ma XD, Lin RS (2016). Mechanism of G9a inhibitor BIX01294 acting on U251 glioma cells. Mol Med Rep.

[38] Kim Y, Lee HM, Xiong Y, Sciaky N, Hulbert SW, Cao X (2017). Targeting the histone methyltransferase G9a activates imprinted genes and improves survival of a mouse model of Prader-Willi syndrome. Nat Med.

[39] Cao H, Li L, Yang D, Zeng L, Yewei X, Yu B (2019). Recent progress in histone methyltransferase (G9a) inhibitors as anticancer agents. Eur J Med Chem.

[40] Liu F, Barsyte-Lovejoy D, Li F, Xiong Y, Korboukh V, Huang XP (2013). Discovery of an in vivo chemical probe of the lysine methyltransferases G9a and GLP. J Med Chem.

[41] Annunziata CM, Davis RE, Demchenko Y, Bellamy W, Gabrea A, Zhan F (2007). Frequent engagement of the classical and alternative NF-kappaB pathways by diverse genetic abnormalities in multiple myeloma. Cancer Cell.

[42] Vallabhapurapu SD, Noothi SK, Pullum DA, Lawrie CH, Pallapati R, Potluri V (2015). Transcriptional repression by the HDAC4-RelB-p52 complex regulates multiple myeloma survival and growth. Nat Commun.

[43] Xie Z, Bi C, Chooi JY, Chan ZL, Mustafa N, Chng WJ (2015). MMSET regulates expression of IRF4 in t(4;14) myeloma and its silencing potentiates the effect of bortezomib. Leukemia.

[44] Li Z, Wong KY, Calin GA, Chng WJ, Chan GC, Chim CS (2019). Epigenetic silencing of miR-340-5p in multiple myeloma: mechanisms and prognostic impact. Clin Epigenet.

[45] Rashid M, Toh TB, Hooi L, Silva A, Zhang Y, Tan PF (2018). Optimizing drug combinations against multiple myeloma using a quadratic phenotypic optimization platform (QPOP). Sci Transl Med.

[46] Andrews S. FastQC: a quality control tool for high throughput sequence data. http://www.bioinformaticsbabrahamacuk/projects/fastqc. 2010.

[47] Subramanian A, Tamayo P, Mootha VK, Mukherjee S, Ebert BL, Gillette MA (2005). Gene set enrichment analysis: a knowledge-based approach for interpreting genome-wide expression profiles. Proc Natl Acad Sci USA.

[48] Reich M, Liefeld T, Gould J, Lerner J, Tamayo P, Mesirov JP (2006). GenePattern 2.0. Nat Genet.

[49] Harrow J, Frankish A, Gonzalez JM, Tapanari E, Diekhans M, Kokocinski F (2012). GENCODE: the reference human genome annotation for The ENCODE Project. Genome Res.

[50] Sun SC (2017). The non-canonical NF-kappaB pathway in immunity and inflammation. Nat Rev Immunol.

[51] Keats JJ, Fonseca R, Chesi M, Schop R, Baker A, Chng WJ (2007). Promiscuous mutations activate the noncanonical NF-kappaB pathway in multiple myeloma. Cancer Cell.

[52] Amodio N, D’Aquila P, Passarino G, Tassone P, Bellizzi D (2017). Epigenetic modifications in multiple myeloma: recent advances on the role of DNA and histone methylation. Expert Opin Ther Targets..

[53] Demchenko YN, Glebov OK, Zingone A, Keats JJ, Bergsagel PL, Kuehl WM (2010). Classical and/or alternative NF-kappaB pathway activation in multiple myeloma. Blood.

[54] Chueh AC, Tse JWT, Dickinson M, Ioannidis P, Jenkins L, Togel L (2017). ATF3 repression of BCL-XL determines apoptotic sensitivity to HDAC inhibitors across tumor types. Clin Cancer Res.

[55] Narita T, Ri M, Masaki A, Mori F, Ito A, Kusumoto S (2015). Lower expression of activating transcription factors 3 and 4 correlates with shorter progression-free survival in multiple myeloma patients receiving bortezomib plus dexamethasone therapy. Blood Cancer J.

[56] Ghosh S, Karin M (2002). Missing pieces in the NF-kappaB puzzle. Cell.

[57] Lauta VM (2003). A review of the cytokine network in multiple myeloma: diagnostic, prognostic, and therapeutic implications. Cancer.

[58] Hanahan D, Weinberg RA (2011). Hallmarks of cancer: the next generation. Cell.

[59] Lowe D, Horvath S, Raj K (2016). Epigenetic clock analyses of cellular senescence and ageing. Oncotarget..

[60] Bali P, Im HI, Kenny PJ (2011). Methylation, memory and addiction. Epigenetics..

[61] Skinner MK (2011). Role of epigenetics in developmental biology and transgenerational inheritance. Birth Defects Res Part C Embryo Today.

[62] Toh TB, Lim JJ, Chow EK (2017). Epigenetics in cancer stem cells. Mol Cancer..

[63] Rodenhiser D, Mann M (2006). Epigenetics and human disease: translating basic biology into clinical applications. CMAJ.

[64] Hu L, Zang MD, Wang HX, Zhang BG, Wang ZQ, Fan ZY (2018). G9A promotes gastric cancer metastasis by upregulating ITGB3 in a SET domain-independent manner. Cell Death Dis.

[65] Hua KT, Wang MY, Chen MW, Wei LH, Chen CK, Ko CH (2014). The H3K9 methyltransferase G9a is a marker of aggressive ovarian cancer that promotes peritoneal metastasis. Mol Cancer..

[66] Kim Y, Kim YS, Kim DE, Lee JS, Song JH, Kim HG (2013). BIX-01294 induces autophagy-associated cell death via EHMT2/G9a dysfunction and intracellular reactive oxygen species production. Autophagy..

[67] Lee JS, Kim Y, Kim IS, Kim B, Choi HJ, Lee JM (2010). Negative regulation of hypoxic responses via induced Reptin methylation. Mol Cell.

[68] Ho JC, Abdullah LN, Pang QY, Jha S, Chow EK, Yang H (2017). Inhibition of the H3K9 methyltransferase G9A attenuates oncogenicity and activates the hypoxia signaling pathway. PLoS ONE.

[69] Béguelin W, Popovic R, Teater M, Jiang Y, Bunting KL, Rosen M (2013). EZH2 is required for germinal center formation and somatic EZH2 mutations promote lymphoid transformation. Cancer Cell.

[70] Wang L, Dong X, Ren Y, Luo J, Yang X (2018). Targeting EHMT2 reverses EGFR-TKI resistance in NSCLC by epigenetically regulating the PTEN/AKT signaling pathway. Cell Death Dis.

[71] Lim J, Goh J, Rashid M, Chow E (2019). Maximizing efficiency of artificial intelligence-driven drug combination optimization through minimal resolution experimental design. Adv Ther.

[72] Vrabel D, Pour L, Sevcikova S (2019). The impact of NF-kappaB signaling on pathogenesis and current treatment strategies in multiple myeloma. Blood Rev.

[73] Demchenko YN, Brents LA, Li Z, Bergsagel LP, McGee LR, Kuehl MW (2014). Novel inhibitors are cytotoxic for myeloma cells with NFkB inducing kinase-dependent activation of NFkB. Oncotarget..

[74] Chen X, El Gazzar M, Yoza BK, McCall CE (2009). The NF-kappaB factor RelB and histone H3 lysine methyltransferase G9a directly interact to generate epigenetic silencing in endotoxin tolerance. J Biol Chem.

[75] Xiao X, Shi X, Fan Y, Wu C, Zhang X, Minze L (2016). The costimulatory receptor OX40 inhibits interleukin-17 expression through activation of repressive chromatin remodeling pathways. Immunity.

[76] Chaturvedi MM, Sung B, Yadav VR, Kannappan R, Aggarwal BB (2011). NF-kappaB addiction and its role in cancer: ‘one size does not fit all’. Oncogene.

[77] Demchenko YN, Kuehl WM (2010). A critical role for the NFkB pathway in multiple myeloma. Oncotarget..

[78] Robak P, Drozdz I, Szemraj J, Robak T (2018). Drug resistance in multiple myeloma. Cancer Treat Rev.

[79] Markovina S, Callander NS, O’Connor SL, Kim J, Werndli JE, Raschko M (2008). Bortezomib-resistant nuclear factor-kappaB activity in multiple myeloma cells. MCR..

