# Supplementary material for: Frequent upregulation of G9a promotes RelB-dependent proliferation and survival in multiple myeloma
Source: Exp Hematol Oncol. 2020 May 22;9:8. doi: 10.1186/s40164-020-00164-4 (PMC7243326; doi:10.1186/s40164-020-00164-4)
Supplement: Supplementary file 1 — Additional file 1. Additional Figures S1-S4; Additional Table S1 and Additional Table S2. [file 40164_2020_164_MOESM1_ESM.pdf]

Additional

Figure S1

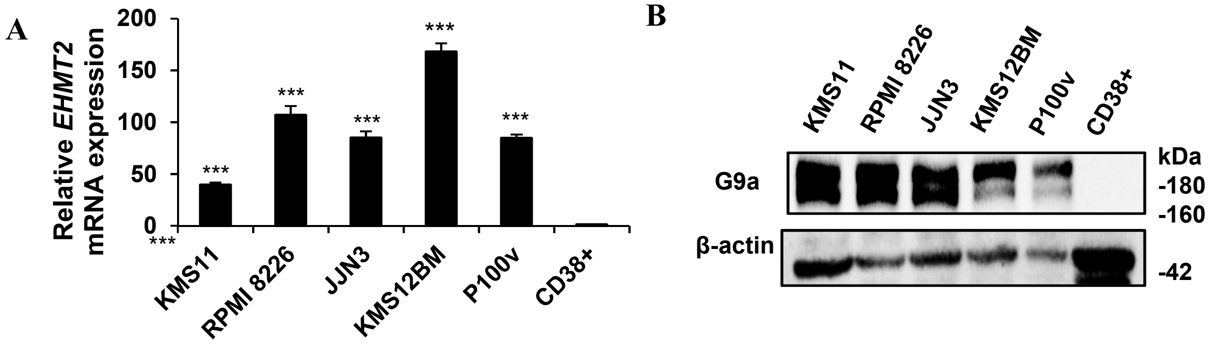

Figure S1. (A) mRNA expression of EHMT2 in multiple myeloma cell lines and CD38 positive ($CD38^+$) PBMC. (n=3; mean ± SD; ***p<0.001). (B) Immunoblots show G9a in MM cell lines and $CD38^+$ PBMC.

Figure S2

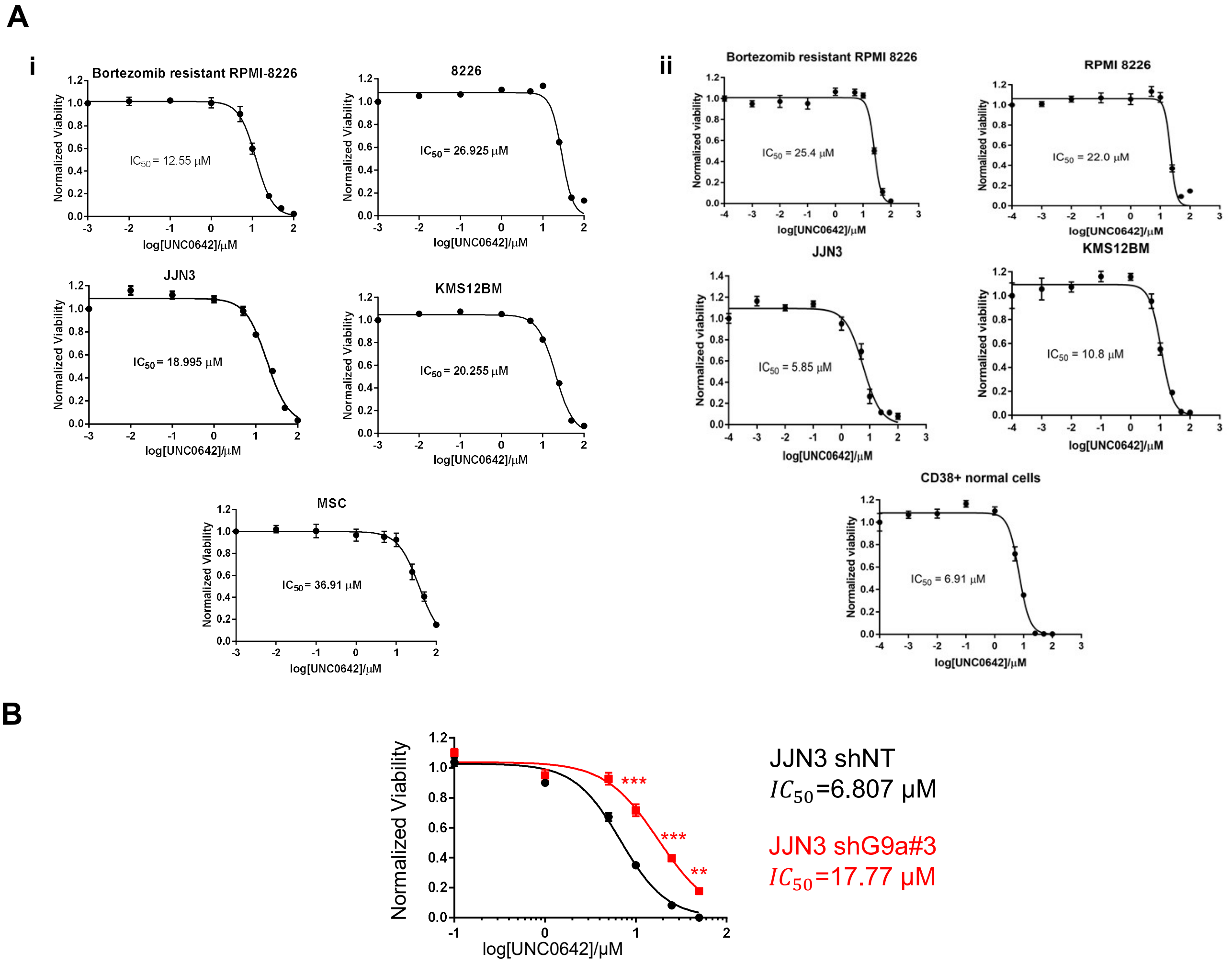

Figure S2.
(A) Dose-response curves and IC50s of UNC0642 on Bortezomib resistant RPMI-8226, RPMI-8226, JJN3, KMS12BM (i) MSC by MTS Cell Proliferation Assay and (ii) $CD38^+$ PBMC by CTG Luminescent Cell Viability Assay. Data are means ± SD. (B) Dose-response curves and IC_{50} of UNC0642 on JJN3 cells infected with lentiviruses expressing shNT or shG9a by CTG Luminescent Cell Viability Assay (n=6; mean ± SD; **p<0.01 and ***p<0.001).

Figure S3

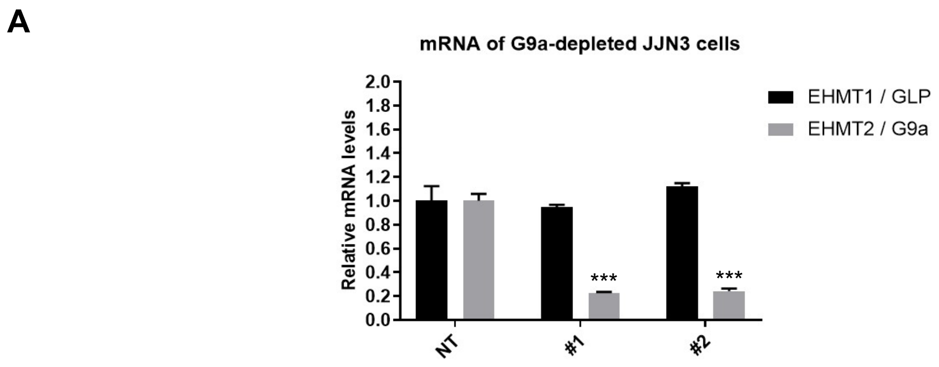

Figure S3.
(A) mRNA expression of EHMT1 and EHMT2 in indicated MM cell lines infected with lentiviruses expressing shNT or shG9a (n = 3; mean ± SD; ***p<0.001).

Figure S4

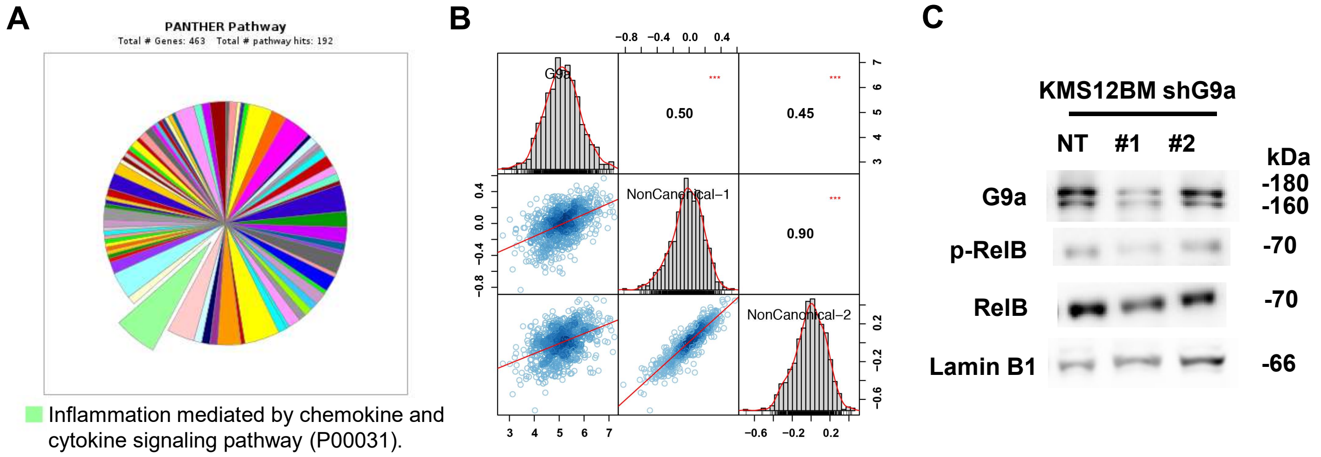

Figure S4.
(A) Pathway analysis using PANTHER pathway database for significantly differential genes of G9a-depleted KMS12BM. (B) Correlations between G9a expression and non-canonical NF-κB signature indices in CoMMpass data. *p < 0.05, **p<0.01, ***p<0.001. (C) Immunoblots show G9a, p-RelB, RelB Lamin B1 as loading control in the nuclear fraction of KMS12BM infected with lentiviruses expressing shNT or shG9a.

Table S1. Patient characteristics by EHMT/G9a expression group

Variable	Top 25%	Middle 50%	Bottom 25%
No. of patients	172	343	172
Sex			
Male	110 (64)	195 (56.9)	105 (61)
Female	62 (36)	148 (43.1)	67 (39)
Age			
Mean (SD)	63.5 (9.69)	63.9 (10.96)	64.1 (11.55)
Median (Q1-Q3)	64 (58-70)	64 (57-71)	64 (56-72)
Minimum-maximum	36-85	27-93	36-90
Race			
Caucasian	137 (79.7)	262 (76.4)	134 (78.4)
African-American	24 (14)	57 (16.6)	25 (14.6)
Other	11 (6.3)	24 (7)	12 (7)
ISS stage			
I	55 (32.9)	109 (32.9)	52 (31.1)
II	53 (31.7)	127 (38.4)	66 (39.5)
III	59 (35.3)	95 (28.7)	49 (29.3)

Table S2. Patient survival characteristics by EHMT/G9a expression group

OS (months)	Top 25%	Middle 50%	Bottom 25%
To event	50	45	22
Mean (SD)	13.4 (8.55)	14.1 (10.88)	17.7 (12.09)
Median (Q1-Q3)	13 (6.7-18.5)	12.2 (5-19.7)	15.5 (8.7-25.5)
Minimum-maximum	0.4-36.5	0.5-48.3	2.1-37.8
To censor	122 (1)[1]	298 (5)	172 (11)
Mean (SD)	22.8 (11.28)	22.8 (11.27)	21.4 (11.33)
Median (Q1-Q3)	22.9 (14.6-30.2)	21.1 (14.1-30.6)	21 (12.4-26.8)
Minimum-maximum	-0.7-51.9	-0.2-49.4	0.2-51.7

PFS (months)	Top 25%	Middle 50%	Bottom 25%
To event	81	114	41
Mean (SD)	13 (9)	15.8 (11)	15.5 (10.13)
Median (Q1-Q3)	11.3 (5.6-18.2)	13.4 (8.4-20)	14.9 (7.2-23.1)
Minimum-maximum	0.4-43.7	0.5-47.2	2.1-45.4
To censor	91 (6)[1]	229 (12)	131 (13)
Mean (SD)	20.2 (10.23)	20.3 (10.32)	19.7 (10.76)
Median (Q1-Q3)	20.5 (12.2-25.6)	20.2 (11.7-26.2)	19.4 (11.4-51.6)
Minimum-maximum	2.8-45.1	1.2-48.9	1.9-51.6

[1] Number of missing information cases
